# Human cerebellar microstructure with 7T MRI: Spherical b-tensor encoding and super resolution reconstruction

**DOI:** 10.1162/IMAG.a.1304

**Published:** 2026-08-12

**Authors:** Emma J.P. Brouwer, Wietske van der Zwaag, René in ‘t Zandt, Nikos Priovoulos, Filip Szczepankiewicz, Markus Nilsson, Karin Markenroth Bloch

**Affiliations:** Spinoza Centre for Neuroimaging, KNAW, Amsterdam, The Netherlands; Netherlands Institute for Neuroscience, Amsterdam, The Netherlands; Lund University Bioimaging Center, Lund University, Lund, Sweden; Biomedical Engineering and Physics, Amsterdam UMC, Amsterdam, The Netherlands; Medical Radiation Physics, Clinical Sciences Lund, Lund University, Lund, Sweden; Diagnostic Radiology, Clinical Sciences Lund, Lund University, Lund, Sweden

**Keywords:** cerebellum, spherical b-tensor encoding, super resolution reconstruction, 7T, granular layer

## Abstract

Cerebellar function and structure remain less studied compared with the cerebrum, and while high-resolution 7T gives superior anatomical information, methods to visualise its microstructure in vivo are not widely available. However, improved understanding of cerebellar microstructure is highly clinically relevant: the granular layer of the cerebellum, located in the grey matter, contains the majority of the brain’s neurons and has been shown in post mortem studies to be affected by various diseases. The limited spatial resolution of MR does not resolve individual cell layers, but advanced diffusion techniques can infer properties of the microstructure. The aim of this work was to implement a combination of spherical b-tensor encoding using free waveforms, $B_{1}^{+}$ field shimming and super-resolution reconstruction (SRR) at 7T and evaluate the method in healthy volunteers. Diffusion-weighted signals and normalised diffusion signals were compared between the grey and white matter of the cerebellum and the occipital lobe as well as across 30 smaller ROIs in the cerebellum. The normalised diffusion signal obtained with spherical tensor encoding relates to cell density, and was found to be increased in the cerebellar cortex relative to the occipital cortex, consistent with previous findings. In the cerebellar cortex, the normalised diffusion signal followed a pattern similar to the thickness of the granular layer reported post mortem. Specifically, increased normalised diffusion signal values were found in lobules VII–VIII and Crus I/II, while lower values were found in lobules I–V. We successfully implemented spherical tensor encoding and SRR at 7T to image the human cerebellum, showing consistent results with those obtained at 3T, with an increased normalised diffusion signal in the cerebellum relative to that found in the cerebral cortex.

## 1 Introduction

The cerebellum is a complex brain structure which is affected in several diseases such as Alzheimer’s disease, Parkinson’s disease, cerebellar hypoplasia, and spinocerebellar ataxias, yet its function, structure, and microstructure remain understudied (Fukutani et al., 1996; Manto, 2005; ten Donkelaar et al., 2023). The cerebellar cortex has a much higher neuronal cell density than the cerebral cortex. In addition, densely foliated structure of the cerebellar cortex (Sereno et al., 2020) makes it difficult to study (Priovoulos & Bazin, 2023). Approximately 80% of all neurons in the brain are located in the cerebellum, the majority of which are found in the granular cell layer: composed of tightly packed granular cells (Giovannucci et al., 2017; Zheng et al., 2022).

Cerebellar imaging benefits from the high SNR available at 7T, which can be traded for spatial resolution in order to resolve the thin and highly foliated cerebellar cortex (Marques et al., 2010; Priovoulos et al., 2023). Ultra-high-field MRI, in combination with $B_{1}^{+}$ shimming, has been employed successfully to capture the cerebellar structure (Priovoulos et al., 2023) and function (Brouwer et al., 2024; van der Zwaag et al., 2013) in vivo. $B_{1}^{+}$ shimming employs phase shims to adjust the $B_{1}^{+}$ field channelwise, increasing or attenuating the field in specific brain regions such as the cerebellum (Brouwer et al., 2025). Specifically the cerebellar surface has been reconstructed with high precision in vivo at 7T (0.4 and 0.2 mm isotropic resolution) (Marques et al., 2010; Priovoulos et al., 2023) as well as post mortem at 9.4T (0.2 mm isotropic resolution) (Sereno et al., 2020). Although 7T-MRI offers increased spatial resolution in these structural and functional imaging methods, this has been more difficult to achieve in diffusion-weighted imaging. The *B*_1_-inhomogeneity associated with ultra-high-field MRI particularly affects the spin-echo efficiency (Norris et al., 2011), and the short T2* values of grey matter at 7T necessitate very short echo times which are difficult to achieve in diffusion-weighted imaging (Feinberg et al., 2023). Despite these challenges, it is beneficial to obtain good quality diffusion-weighted data also at 7T as microstructural information can then be acquired alongside high-resolution structural and functional data.

### 1.1 Post mortem studies of the cerebellar granular layer

Knowing the thickness and density of the granular layer of the cerebellum is crucial to understanding its role in diseases associated with cerebellar dysfunction as well as its structure in healthy controls. Although microscopic resolutions of the brain cannot be acquired in vivo, this can be achieved in post mortem studies. Such histological studies that investigate the cerebellar granular layer in humans are scarce but some examples exist. For instance, Kalanjati et al. (2017) used histological staining to show that the granular cell nuclei can be observed at a magnification of 400x. Zheng et al. (2022) have provided a detailed 3D reconstruction of the cerebellar granular layer from a human brain sample at 20 µm isotropic resolution, showing that the granular layer is thicker in lateral–posterior–inferior cerebellar regions (Lobules VI–VIII and Crus I/II) than medial-superior regions (I–IV and Vermis) in healthy controls. Post mortem histological studies have also provided insights into granular cell layer pathology across various conditions and diseases. For example, Canedo Pouso (2022) showed that COVID-19 patients have a lower granular cell count in the cerebellum than healthy controls. Cerebellar hypoplasia has been shown to cause degeneration of the cerebellar granular layer (Norman, 1940; ten Donkelaar et al., 2023). In Alzheimer’s disease, reductions in granular layer volume (Wegiel et al., 1999) and granular layer thickness (Andersen et al., 2012) have been reported. In contrast, a study on Parkinson’s disease reported no difference in granular cell count between patients and controls (Rusholt et al., 2020). These findings underscore the uncertainty of how the various diseases affect the granular layer. Furthermore, since granular cell counts are performed post mortem, the limited availability of samples hinders the investigation of granular layer pathology. Therefore, in vivo methods would provide substantial benefits for studies of cerebellar microstructure.

### 1.2 Spherical b-tensor encoding and super resolution reconstruction

Within the small granular cells, diffusion is restricted in all directions, making diffusion MRI a promising tool to study the distribution of the granular cell layers in humans in vivo. A tentative marker of granular cells is the so-called dot-like signal, which refers to the signal observed at very high b-values that arises from regions where diffusion is highly restricted in all directions (Alexander et al., 2010; Tax et al., 2020). It can be calculated by dividing the diffusion signal at high b-values (approximately 7000 s/mm^2^ and higher), (Tallantyre et al., 2011) by the image signal without diffusion weighting (b-value of 0 s/mm^2^). However, it is challenging to separate the dot fraction from the restricted diffusion of water within thin axons running perpendicular to the encoding direction, which are prevalent as axons often display a high orientation disorder. A solution to this problem is to use isotropic diffusion weighting schemes which were introduced in the 1990s (Mori & Van Zijl, 1995; Wong et al., 1995) and have seen increasing implementation and development in recent years (Lasič, Szczepankiewicz, et al., 2020; Lasič, Lundell, et al., 2020; Nilsson et al., 2018, 2021; Szczepankiewicz, Sjölund, et al., 2019). Specifically, so-called spherical b-tensor encoding (Szczepankiewicz, Westin, et al., 2019), which is a method in which gradients are applied in three orthogonal directions during the diffusion encoding, has been used to map the dot-like signal (Tax et al., 2020). Spherical tensor encoding is sensitive to the isotropic, densely packed cellular environment typical of granule cells, making it a promising tool to capture these. However, a drawback of spherical tensor encoding is that its diffusion encoding is less efficient than conventional encoding (Szczepankiewicz et al., 2021), which leads to longer echo times that consequently results in lower signal-to-noise ratio (SNR). This, in turn, necessitates the use of a method to improve the SNR. This can be achieved by so-called super-resolution reconstruction (SRR) where multiple low-resolution images are acquired in different orientations and reconstructed into a higher resolution image (Shilling et al., 2009; Vis et al., 2021). Previous work using spherical tensor encoding and SRR at 3T has revealed differences in the dot-like signal distribution between cerebellar and cortical grey matter (Vis et al., 2021) and differences in the cerebellar grey matter between patients with movement disorders (Dystonia and Spinocerebellar Ataxia type 6) and healthy controls (Tax et al., 2023). These variations are potentially driven by differences in the granular cell layer structure in the cerebellum.

The aim of this work was to implement a combination of spherical tensor encoding at 7T using free waveforms, cerebellar *B*1^+^ field shimming and SRR. In order to compare our results with recent post-mortem estimations of the granular layer thickness of the cerebellar surface, we assessed diffusion signal differences across cerebellar lobules and reconstructed the cerebellar surface of one participant to provide a direct comparison in the 3D surface space.

## 2 Methods

### 2.1 Participants

In total, 11 participants (7 females, 21–51 years) were scanned using an actively shielded 7T Achieva scanner (Philips, Best, the Netherlands) with an 8Tx/32Rx head coil (Nova Medical, Wilmington, MA, USA). All participants provided written informed consent as approved by the local ethics committee.

### 2.2 Acquisition parameters

**Fig. 1.**
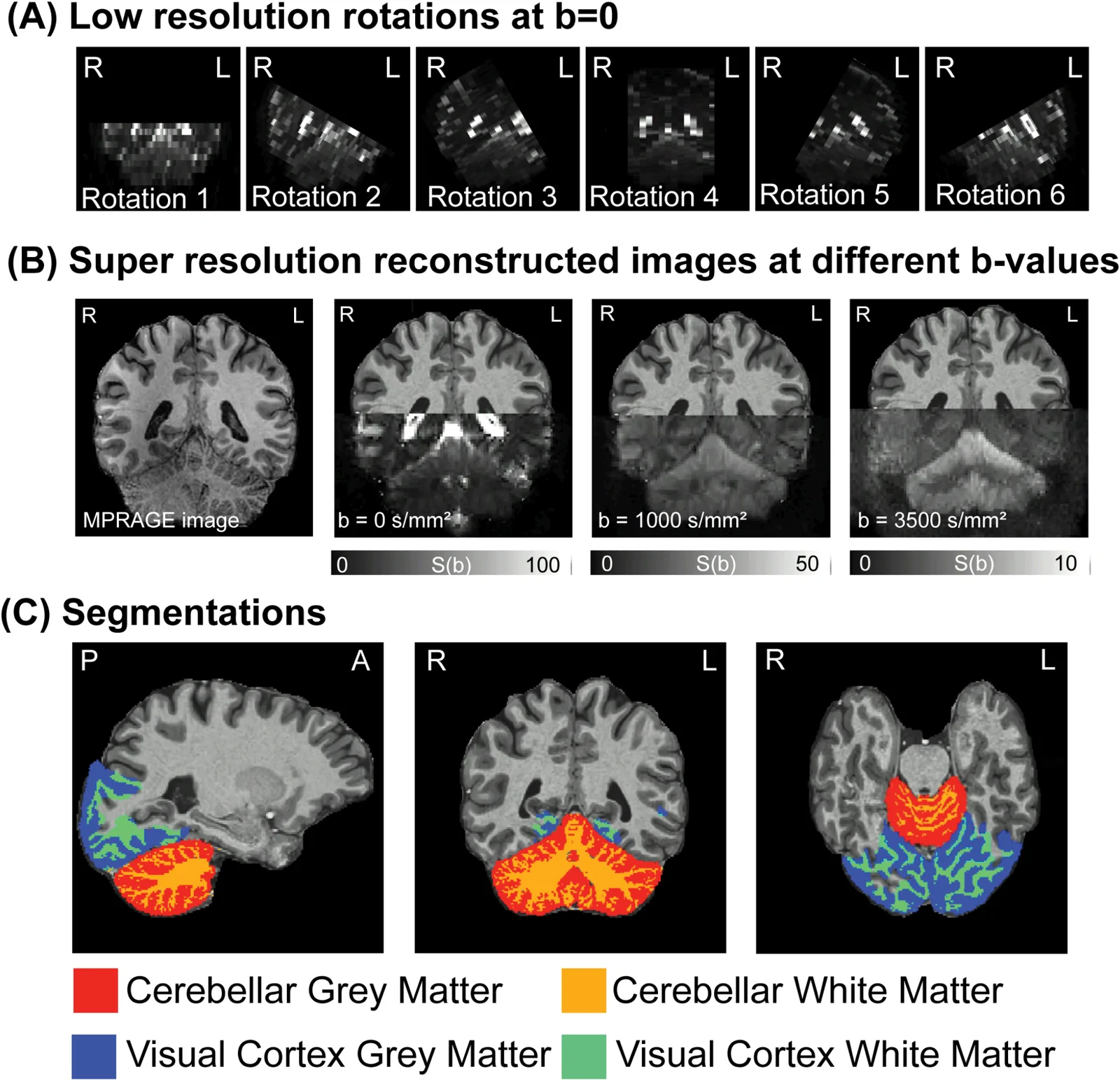
Acquisition field of view example from participant 1. (A) The low-resolution (1.7 × 1.7 × 7.0 mm^3^) EPI (0 s/mm^2^) for each of the 6 rotations with increment of 30°. (B) The super-resolution reconstructed high-resolution images (1.7 × 1.7 × 1.7 mm^3^) including the upper half of the MPRAGE image which is not covered by the diffusion-weighted image for all three b-values (left: 0 s/mm^2^, middle: 1000 s/mm^2^, right: 3500 s/mm^2^). (C) Sagittal, coronal, and axial views of the cerebellar and visual cortex grey/white matter segmentations overlaid onto the MPRAGE image.

Diffusion MRI was performed using a custom spin echo sequence with EPI readout sequence with user-defined diffusion gradient waveforms (Szczepankiewicz, Sjölund, et al., 2019). The gradient waveforms were optimised for spherical b-tensor encoding using the NOW framework (Sjölund et al., 2015; Szczepankiewicz, Sjölund, et al., 2019), for a maximum gradient amplitude of 60 mT/m. The waveforms are shown in the Supplementary Figure S4. The cerebellum was ${\text{B}}_{1}^{+}$ shimmed using MRcodeTool (Tesla Dynamic Coils, Zaltbommel, NL). Detailed imaging parameters of all performed acquisitions are given in Table 1. A slab with high in-plane (axial) resolution but thick slices (1.7 × 1.7 × 7 mm^3^) was defined covering the cerebellum and the occipital lobe and rotated around the anterior–posterior axis in 6 steps of 30^°^ increments resulting in a total scan time of 7 minutes for all rotations (Fig. 1A), akin to methods presented by Vis et al. (2021). This acquisition was performed three times for all participants to assess stability. Between these runs, data were acquired with opposite phase-encoding directions in the anterior–posterior axis to allow for distortion correction using topup (Jenkinson et al., 2012). For each participant, an MPRAGE image (1 mm isotropic) was acquired for image registration and segmentation of grey and white matter fractions in the cerebellum and occipital lobe.

**Table 1.** Overview of sequence acquisition parameters.

	STE-EPI	MPRAGE	MP2RAGE
Field of view (mm^3^)	220 × 220 × 98	200 × 252 × 190	210 × 210 × 60
Acquired resolution (mm^3^)	1.7 × 1.7 × 7.0	1.0 × 1.0 × 1.0	0.4 × 0.4 × 0.4
Reconstructed resolution (mm^3^)	1.7 × 1.7 × 1.7	–	–
TE (ms)	100	1.9	1.9
TR (ms)	5000	5.0	5.7
b-values (s/mm^2^)	[0, 1000, 3500]	–	–
b-value repetition	[3, 4, 7]	–	–
Number of participants	11	11	1
Number of runs	3	1	1

Additionally, for one participant (#9), a sub-millimetre (0.4 mm isotropic) prospective motion-corrected MP2RAGE image of the cerebellum was acquired, in line with methods presented by Priovoulos et al. (2023) (Table 1) to generate a 3D cerebellar surface rendering for comparison with the granular layer thickness presented by Zheng et al. (2022).

### 2.3 Data analysis

Low-resolution spherical tensor-encoded images were denoised using ANTs v2.6.2 (Avants et al., 2009), motion corrected using FSL v6.0.7 (Jenkinson et al., 2012) and averaged across b-value repetitions. For each of the three runs, images were reconstructed using SRR frameworks (Vis et al., 2021), available in open source at https://github.com/filip-szczepankiewicz/Vis_NIMG_2021/, to a resolution of 1.7 mm isotropic (regularisation factor = λ=0.05 ) (Fig. 1B). These high-resolution images were distortion corrected using topup and registered to the participant anatomical space for further analysis (Avants et al., 2009; Jenkinson et al., 2012).

The grey and white matter (GM, WM) in the cerebellum and visual cortex were segmented from the MPRAGE image using SUIT (Diedrichsen, 2006) and SPM12 (Ashburner & Friston, 2005) (Fig. 1C). A cerebellar lobule mask (consisting of 30 cerebellar ROIs) (Diedrichsen, 2006) and a visual cortex mask were non-linearly registered to each participant’s space (Avants et al., 2009) to allow for comparisons within these regions.

To assess stability of the spherical tensor-encoded-EPI acquisitions across runs, a Pearson correlation between the median diffusion signal value within segmentations was performed across b-values. Next, to establish the unique contrast between the cerebellum and the cerebral cortex, the diffusion-weighted signal was compared across white and grey matter segmentations in the cerebellum and the visual cortex by taking the median diffusion-weighted signal across runs and participants within each segmentation. A repeated measures ANOVA was performed to compare signal intensities across segmentations, participants, and b-values using JASP Team (2023).

A **normalised diffusion signal** was calculated across participants for a b-value of 1000 and 3500 s/mm^2^ in the following manner:

$$f_{norm}=\frac{f\left(b\neq 0s/mm^{2}\right)}{f\left(b=0s/mm^{2}\right)},$$ (1)

where b ≠ 0 s/mm^2^ is the diffusion-weighted signal at either a b-value of 1000 s/mm^2^ or 3500 s/mm^2^ (Tax et al., 2020, 2023). In this study, we refer to these values as the “normalised diffusion signal” instead of the “dot-like signal” because the gradient strength of the scanner did not allow for sufficiently high b-values to ensure that only the dot fraction contributed to the signal (Chakwizira et al., 2023). We first compared the normalised diffusion signal of gray matter in the cerebellum and in the visual cortex, expecting a higher *f_dot_* in the cerebellar gray matter as has been shown for 3T (Tax et al., 2023; Vis et al., 2021). Next, the median-normalised diffusion signal (for b = 3500 s/mm^2^) was plotted across 30 cerebellar ROIs drawn from the SUIT atlas (Diedrichsen et al., 2009). These values were correlated to the granular layer thickness obtained in these ROIs by Zheng et al. (2022). Finally, white and grey matter were segmented in the 0.4 mm isotropic MP2RAGE image of one participant using a cerebellar-specific segmentation algorithm (Priovoulos & Bazin, 2023). These segmentations were used to create a cerebellar surface reconstruction using Nighres (Huntenburg et al., 2018). The normalised diffusion signal of a single participant and run was projected onto the 3D surface space using Paraview (Ahrens et al., 2005) in order to compare the signal pattern with the 3D surface reconstruction of the granular layer thickness presented by Zheng et al. (2022).

## 3 Results

### 3.1 Diffusion-weighted signal across b-values

The spherical tensor-encoded-EPI-free waveform sequence with SRR was successfully implemented at 7T, resulting in 1.7 mm isotropic reconstructed images with full coverage of the cerebellum for three different b-values (0, 1000, and 3500 s/mm^2^) (Fig. 1A, B). The resulting diffusion-weighted signals were consistent across three runs with an average Person correlation of *r* = 0.86, *r* = 0.88, and *r* = 0.78 for b-values of 0, 1000, and 3500 s/mm^2^, respectively (Supplementary Figs. S1 and S2).

**Fig. 2.**
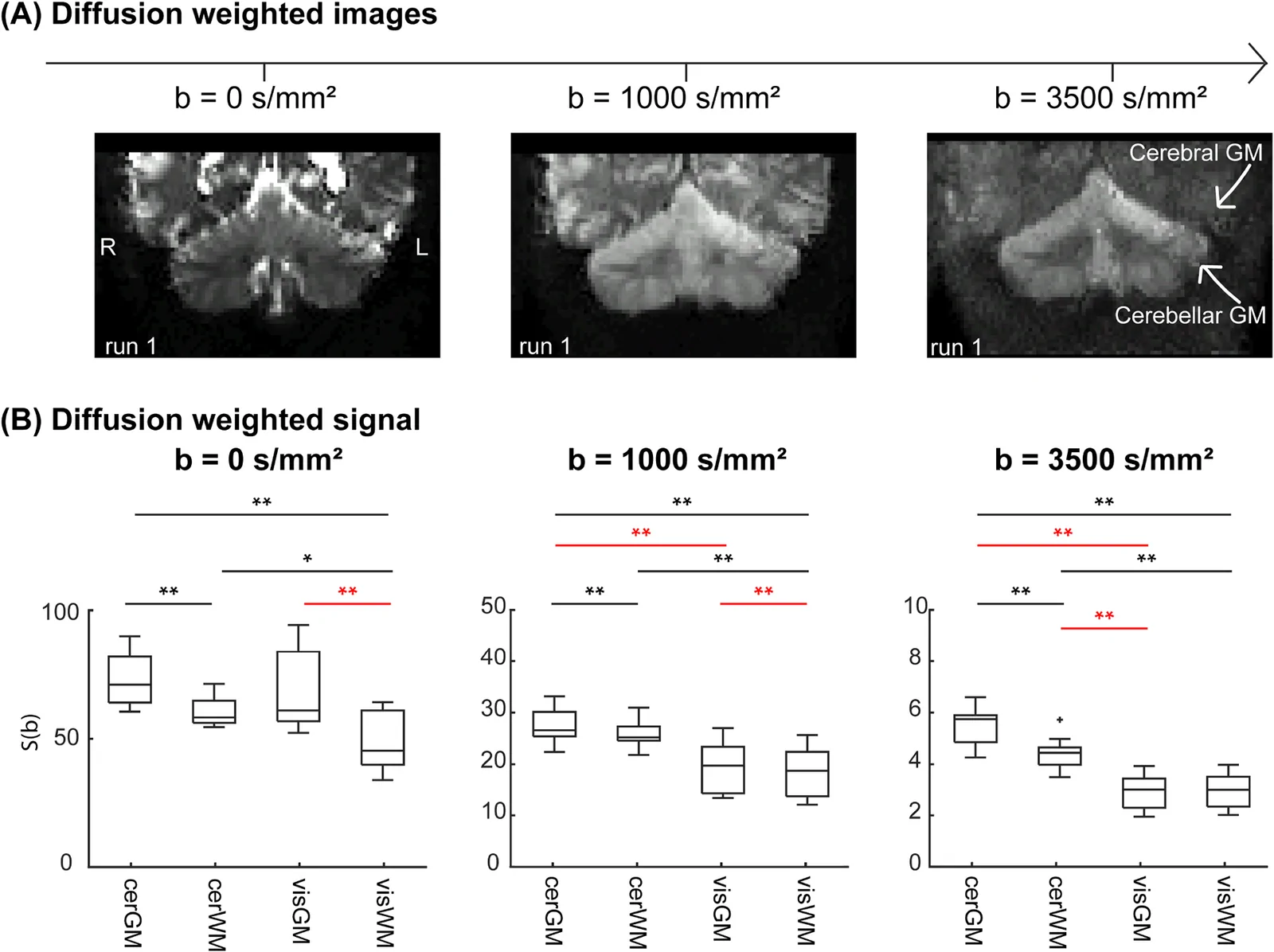
(A) A coronal view of the diffusion-weighted signal across b-values (left: 0 s/mm^2^, middle: 1000 s/mm^2^, right: 3500 s/mm^2^) for participant 1. Note how the contrast between the cerebral cortex and the cerebellar grey matter becomes larger at higher b-values. (B) The diffusion-weighted signal, S(b), across b-values, averaged over runs 1–3 (left: 0 s/mm^2^, middle: 1000 s/mm^2^, right: 3500 s/mm^2^) for cerebellar grey matter (cerGM), cerebellar white matter (cerWM), visual cortex grey matter (visGM), and visual cortex white matter (visWM). Significant differences are indicated with a red line if they do not occur across all b-values. Note that at a b-value of 3500 and 1000 s/mm^2^ there is a significant difference between the cerebellar GM and the visual cortex GM. At a b-value of 3500, the contrast between the visual cortex GM and WM is lost, indicating dominance of the diffusion weighting over the T2 weighting of the EPI. Asterisks indicate statistical significance: *p < 0.05, **p < 0.01.

Across data from 3 runs and 11 participants, grey and white matter signal intensities were similar in the visual cortex and the cerebellum when using a b-value of 0 s/mm^2^, signalling appropriate image intensity homogeneity using the cerebellar $B_{1}^{+}$ shim (Fig. 2; Supplementary Table S1). Both grey matter compartments had higher signal intensity than the white matter compartments, in agreement with the longer T2 values in the grey matter and the T2 weighting of the EPI images. In contrast, diffusion-weighted signal intensities in the grey and white matter were higher in the cerebellum than in the visual cortex using b-values of 1000 and 3500 s/mm^2^ (Fig. 2; Supplementary Tables S2 and S3). This is also shown in Figure 2 (for b = 3500 s/mm^2^) where the cerebellar grey matter appears brighter than that of the visual cortex. This contrast is larger using a b-value of 3500 s/mm^2^ than using 1000 s/mm^2^ (Supplementary Tables S2 and S3). At a b-value of 3500 s/mm^2^, the contrast between the grey and white matter in the visual cortex is almost fully lost, whereas in the cerebellum, the grey and white matter contrast remains present across b-values (Fig. 2B, Supplementary Table S5), such that at a b-value of 3500 s/mm^2^, only the cerebellar grey matter compartment remains bright.

### 3.2 Normalised diffusion signal

**Fig. 3.**
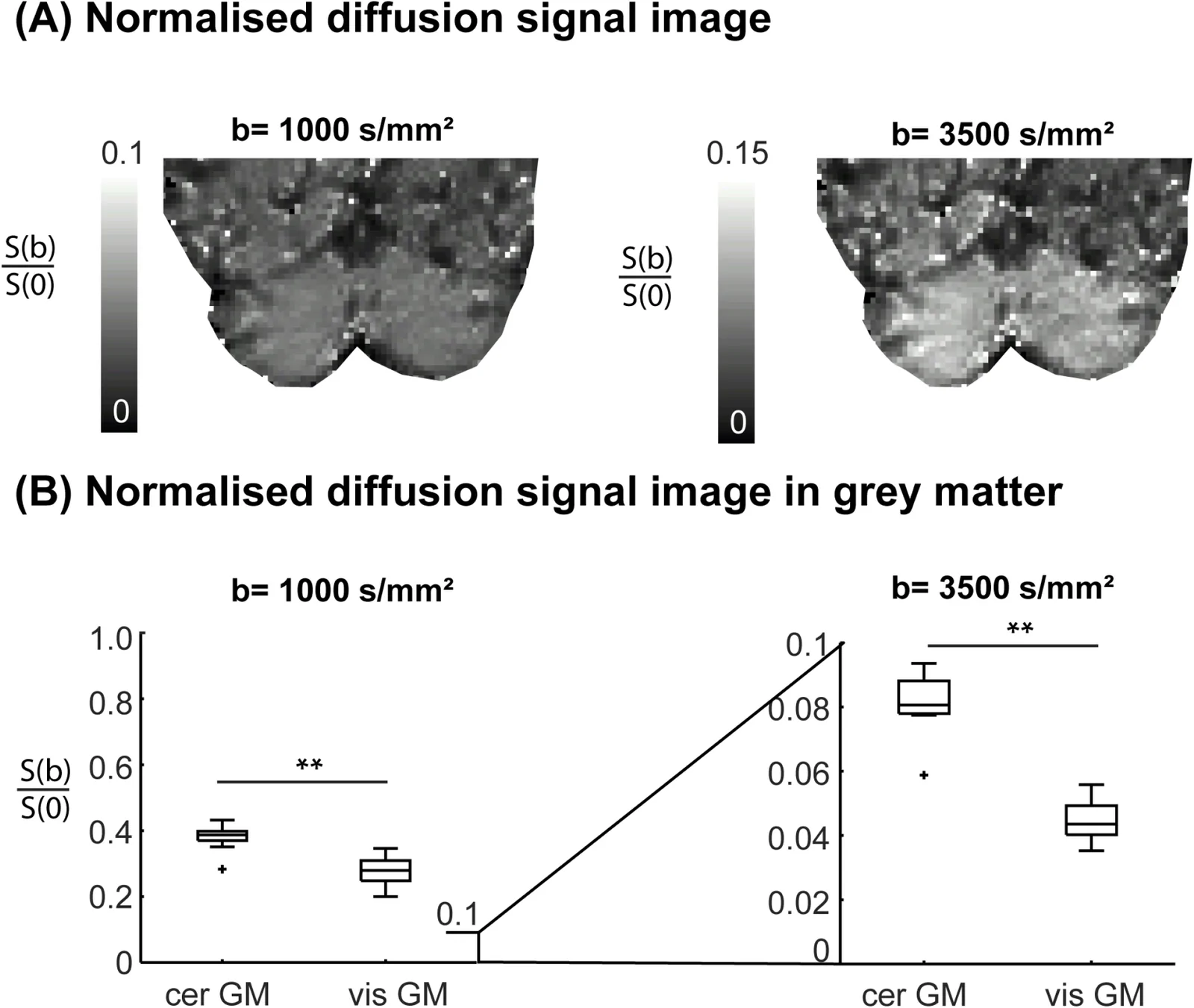
(A) An example image of the normalised diffusion signal for participant 1, left: *s*(*b* = 1000), right: *s*(*b* = 3500). (B) The median-normalised diffusion signal of cerebellar grey matter (cer GM) and visual cortex grey matter (vis GM), averaged over runs 1–3 for $fnorm=S(b=1000s/mm^{2})/S(b=0s/mm^{2})$ (left) and for $fdot=S(b=3500s/mm^{2})/S(b=0s/mm^{2})$ (right). ** indicates p<0.001 for a paired t-test.

Similar to the diffusion-weighted signal, values of the normalised diffusion signal (Fig. 3) are significantly higher in cerebellar grey matter than in the occipital grey matter (*p* < 0.001, *d* = 3.94; Supplementary Tables S5 and S6).

**Fig. 4.**
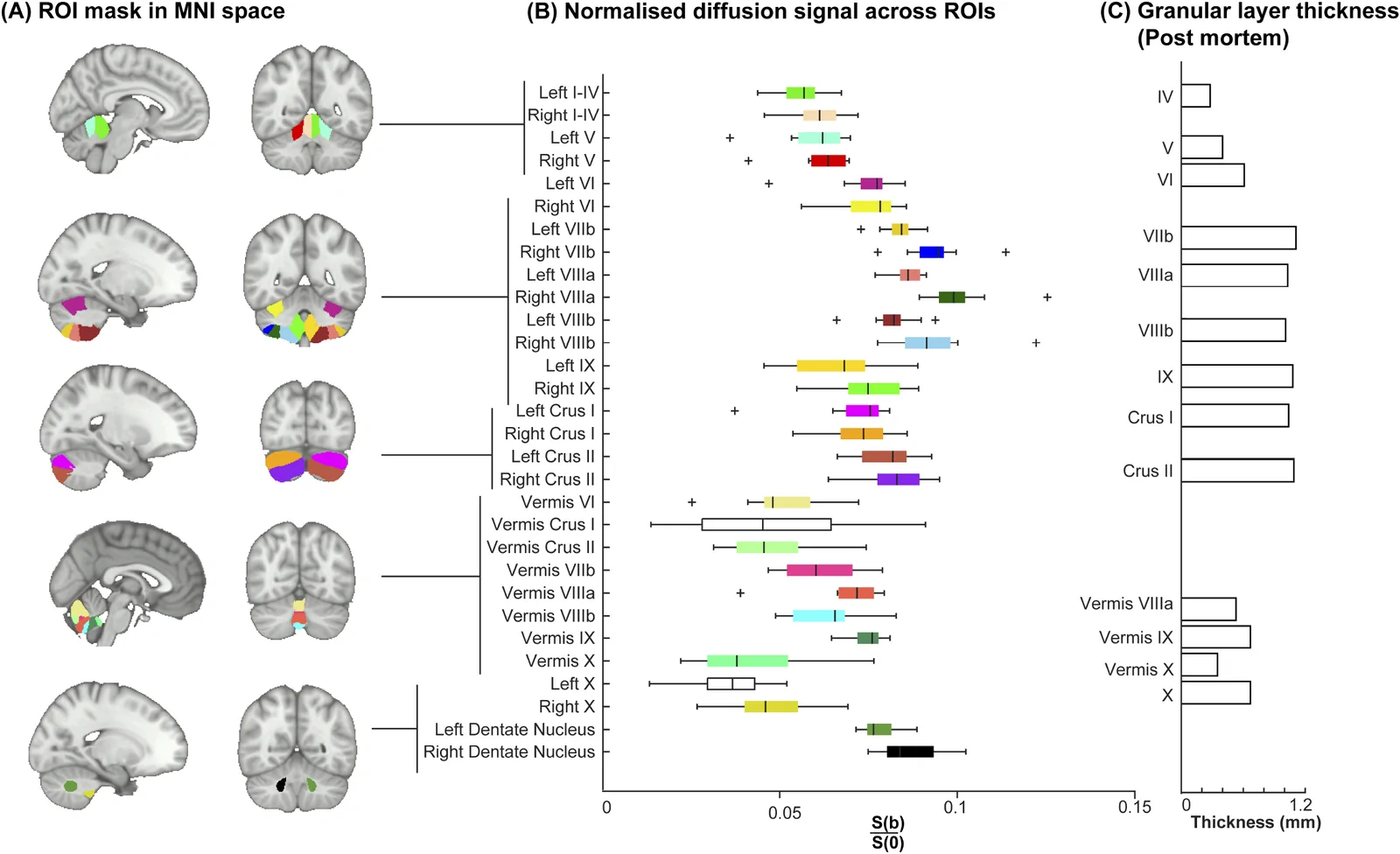
(A) The lobule ROIs from (B) presented in MNI space in the sagittal and axial planes. (B) The median-normalised diffusion signal of the cerebellum averaged over runs 1–3 for $S(b=3500s/mm^{2})/S(b=0s/mm^{2})$ across 30 different cerebellar ROIs, outliers are plotted individually using the “+” marker symbol. (C) The granular layer thickness of several lobules, data obtained from Zheng et al. (2022).

Subsequently, we explored how the normalised diffusion signal differed across cerebellar ROIs. In lobules I–V (anterior lobe), normalised diffusion signals were lower than those in lobules VI–IX (posterior lobe) (Fig. 4). Normalised diffusion signals were also lower in the vermis and lobule X than those in the CRUS I/II (Fig. 4). In the dentate nucleus, normalised diffusion signals were similar to those in Crus I/II (Supplementary Table S7). Between participants, the standard deviation of the normalised diffusion signal was similar across ROIs (SD ≈ 0.1; Supplementary Table S7).

**Fig. 5.**
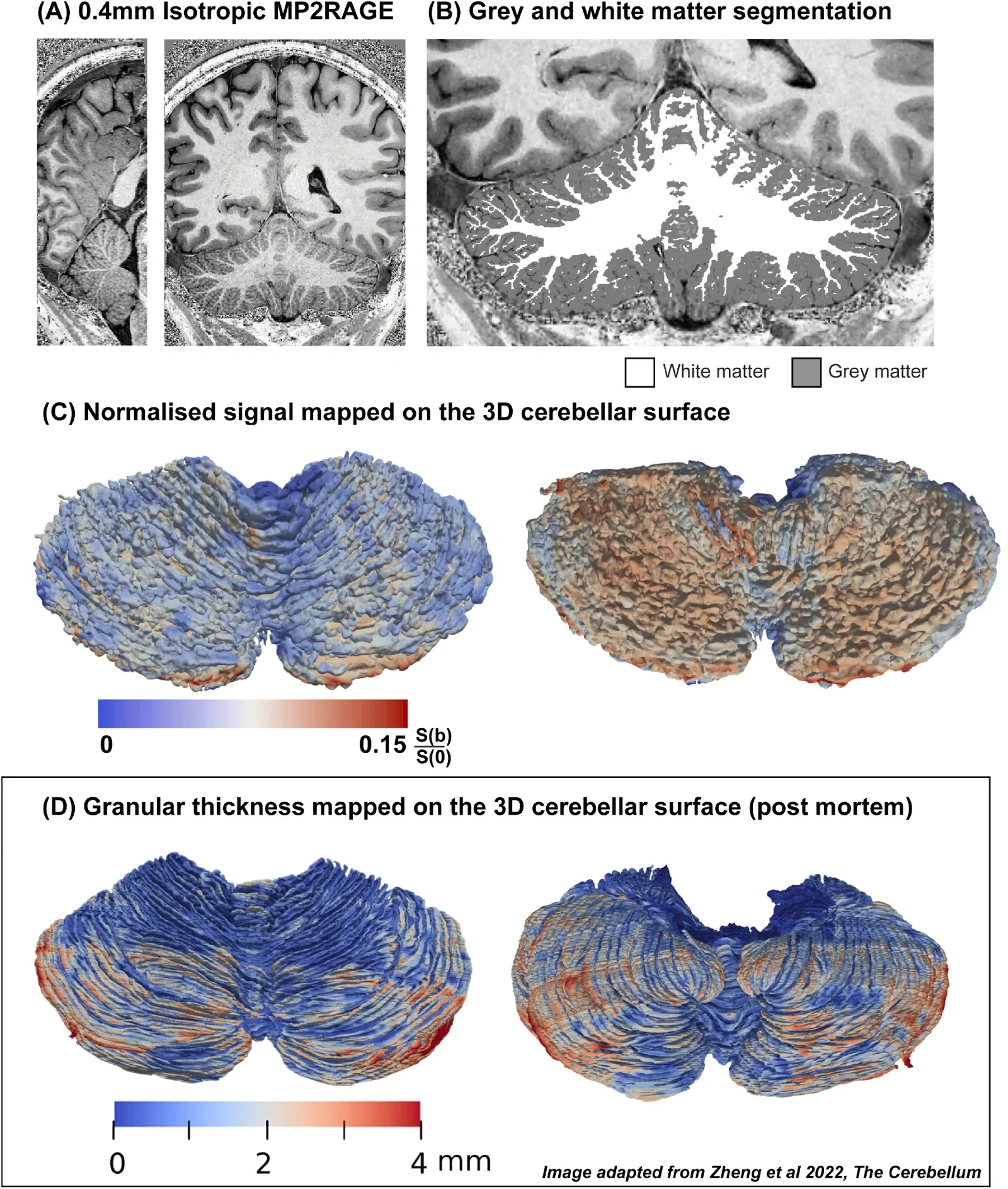
(A) The MP2RAGE image (0.4 × 0.4 × 0.4 mm^3^) used for the cerebellar surface segmentation in (B). (C) The median-normalised diffusion signal of cerebellar grey matter (cer GM) averaged over runs 1–3 $S(b=3500s/mm^{2})/$ $S(b=0s/mm^{2})$ for one participant. Left image: Anterior cerebellar view, right image: Posterior cerebellar view, the left of the surface corresponds to the left of the cerebellum, the right of the surface corresponds to the right of the cerebellum. (D) The granular layer thickness of a single participant adapted from Zheng et al. (2022). Left: Anterior view, right: posterior view. Note in both (C) and (D), values are lower in the anterior lobe view than in the posterior lobe view. Note that on the histological surface (D), the anterior lobe shows more blue (indicating a thinner granular layer) and the posterior lobe more red (indicating a thicker granular layer). A comparable anterior–posterior pattern is observed in our in vivo 3D rendering (C).

Finally, we compared the normalised diffusion signal with the histological findings from a post mortem study by Zheng et al. (2022). There is a strong positive correlation (r=0.72,p<0.006 ) between the normalised diffusion signal (Fig. 4; Supplementary Fig. S3) and the granular thickness measures (Zheng et al., 2022). In contrast, the correlation between the normalised diffusion signal and the thickness of the molecular layer is not significant (r=0.51,p=0.07 ) (Zheng et al., 2022) (Supplementary Fig. S3). The positive correlation between the granular thickness and the normalised diffusion signal measures is further illustrated by the 3D cerebellar surface mapping: the normalised diffusion signal mapped onto the 3D surface (Fig. 5C) resulted in a similar pattern (higher signal values in the posterior view than in the anterior view) compared with the 3D surface visualisation portraying granular layer thickness (larger granular layer thickness in the posterior view than in the anterior view) (Fig. 5D; Zheng et al., 2022).

## 4 Discussion

We successfully implemented spherical tensor encoding and SRR at 7T to image the human cerebellum, showing consistent results with those obtained at 3T, with an increased normalised diffusion signal in the cerebellum relative to that found in the cerebral cortex (Tax et al., 2020, 2023; Vis 2021). The application of spherical tensor encoding and SRR at 7T, with $B_{1}^{+}$ field shimming, facilitated a more detailed structural evaluation of the cerebellum than earlier 3T studies, specifically by identifying normalised diffusion signal differences across the lobules of the cerebellum as well as a sub-millimetre 3D surface rendering of the cerebellar surface which allows fast inspection of the normalised diffusion signal over the cerebellar surface.

The majority of spherical tensor encoding diffusion acquisitions in humans are conducted at 3T, where its implementation has been used for tissue identifications, successfully differentiating between tumours such as gliomas and meningiomas (Szczepankiewicz et al., 2016) and demonstrating grey matter differences between the cerebellum and the cortex (Tax et al., 2023). Although 3T MRI typically allows longer TEs and offers higher b-values in diffusion-weighted imaging than 7T, identifying smaller sub-cerebellar regions of interest can be challenging due to the low image SNR at 3T and, consequently, the reduced spatial resolution in the anatomical reference images. Although some implementations of spherical b-tensor encoding at 7T exist (Martin et al., 2020; Szczepankiewicz, Sjölund, et al., 2019), it has not previously been implemented specifically for imaging of the cerebellum. Implementing this method in the cerebellum at 7T enabled the acquisition of high-resolution anatomical images (≈0.4–1.00 mm isotropic) alongside the diffusion acquisitions, thereby allowing a comparison of the normalised diffusion contrast within cerebellar subregions. This also facilitated comparisons between the normalised diffusion signals and post mortem histological results per lobule and on a cerebellar surface rendering, as displayed in Figure 5. If such images were acquired in patient groups with cerebellar damage, the relationships between cerebellar anatomy and microstructure could provide a better understanding of how the granular layer is impacted by neurological diseases in vivo. In addition, using even higher resolution cerebellar anatomical images (≈0.2 mm) to capture the cerebellar layers (Priovoulos et al., 2023), it may be interesting to identify whether the diffusion signal reflects granular cell density or granular cell layer thickness as both thickness and density have shown to vary across different neurological diseases (Canedo Pouso, 2022; ten Donkelaar et al., 2023). Nevertheless, even 200 µm resolution might not be sufficient for granular cell layer thickness measures (Sereno et al., 2020), while higher resolutions may remain out of reach in vivo.

The implementation of spherical tensor encoding in the cerebellum at 7T could facilitate comparisons with other imaging techniques available at this field strength. For instance, 7T fMRI has proven highly beneficial for imaging cerebellar function at a small scale (Brouwer et al., 2024; van der Zwaag et al., 2013). Combining fMRI results with microstructural markers in vivo could, therefore, provide valuable information into cerebellar functions and their relationship with the granular layer. In particular, this study shows lower normalised diffusion signals in lobules I–V and the vermis than those in lobules VI–IX and Crus I/II. Cerebellar fMRI studies also show functional differences in these regions, with the anterior (lobules I–V) and posterior lobe (lobules VI–X) of the cerebellum involved in different cognitive and motor functions (King et al., 2019; Nettekoven et al., 2024). Lesions in lobules I–V of the anterior lobe in stroke patients have been linked to motor deficits, while those in lobules VII–X of the posterior lobe typically result in minor or no motor deficits, primarily affecting cognitive function (Schmahmann et al., 2009). A meta-analysis of functional cerebellar studies indicates that lobules VI, VII, and Crus I are involved in working memory and executive function tasks (Stoodley & Schmahmann, 2009). Additionally, studies using sensorimotor task paradigms report functional organisational differences between the motor regions of the anterior and posterior lobes (Brouwer et al., 2024; Grodd et al., 2001; van der Zwaag et al., 2013). Hence, it could be interesting to combine diffusion and functional MRI to explore how regional differences in the granular layer may relate to cerebellar functional organisation.

Nevertheless, it is essential to consider the drawbacks that accompany the implementation of spherical diffusion-encoded imaging at 7T. First, at higher field strengths $B_{1}^{+}$ inhomogeneities typically cause substantial signal loss in the cerebellum. In the current study, we minimised these by using individually optimised $B_{1}^{+}$ shims necessitating acquisition of *B*_1_-maps and online calculation of phase offsets. Scan time could be reduced if we were to use group-optimised shimming methods without the need for individual optimisation during the scan session. Second, even though our results reveal differences in the normalised diffusion signal that are in line with histological findings by Zheng et al. (2022), we cannot assert that our normalised diffusion signal is exclusively capturing properties of the cerebellar granular layer. Given the used spatial resolution of 1.7 mm isotropic, which is larger than the thickness of the cerebellar layers and folds, all voxels will also include signals resulting from other compartments such as Purkinje cells, the molecular layer, and white matter. However, we believe it is likely that our normalised diffusion signal is dominated by the cerebellar granular cells as the spherical tensor encoding signal is dominated by signal from water in structures that allow low diffusivity. The granular layer is characterised by small and particularly dense cells, resulting in restricted diffusion in all directions. Furthermore, dendritic spines could also contribute to reduced diffusion signal. However, the extent of this effect is influenced by the gradient waveform and its capacity to encode for exchange (Chakwizira et al., 2023; Lauren Jantzie, 2025). In order to confirm that our diffusion signal is capturing the cerebellar granular layer properties, higher b-values could help to ensure that only the dot fraction contributed to our measured signal. However, since clear contrast between cerebellar and cerebral grey matter is visible in our data using a b-value of 1000 and 3500 s/mm^2^, higher b-values would not necessarily provide additional benefits, also given the associated SNR loss this would incur using our current hardware set-up. Additionally, higher in-plane resolution could help to reduce the partial volume effects. Both higher b-values and increased in-plane resolution would be feasible with the use of 7T MRI scanners with higher gradient strengths or the use of insert gradients in the current 7T MRI system (Feinberg et al., 2023; Vachha & Huang, 2021; Versteeg et al., 2021).

To conclude, imaging the structural properties of the cerebellum in vivo remains challenging due to its thin and highly foliated cortical sheet and because the spatial resolution of MRI is insufficient to capture the individual granular, molecular, and Purkinje cell layers in vivo. Advanced techniques such as spherical tensor encoding dMRI with SRR offer a unique contrast that might provide complementary information about the complex microstructure of the granular layer in the cerebellum. We demonstrated that this method can be successfully applied on a 7T scanner system with 60 mT/m gradients, enabling combinatorial use of high-resolution anatomical imaging and complementary microstructure imaging. This allows for maximal benefit from the unique contrast, supports comparisons at the sub-cerebellar level, and enables surface visualisations similar to those found in post-mortem studies.

## Supplementary Material

Supplementary Material

## Data Availability

Due to ethical restrictions and participant privacy concerns, the neuroimaging data are not publicly available. Anonymised data can be accessed upon request by qualified researchers via the corresponding author subject to a signed Data Access Agreement. The SRR framework is available on https://github.com/filip-szczepankiewicz/Vis_NIMG_2021/.

## References

[IMAG.a.1304-b23] Aggarwal, M. & Jantzie, L. (2025). Disentangling microscopic anisotropy and diffusion time dependence in the brain using oscillating gradients with spherical b-tensor encoding. In Proceedings of the 2025 ISMRM & ISMRT Annual Meeting and Exhibition. Honolulu, HI, USA. Abstract number: 130. 10.58530/2025/0130

[IMAG.a.1304-b1] Ahrens, J., Geveci, B., & Law, C. (2005). ParaView: An end-user tool for large-data visualization. In C. D. Hansen & C. R. Johnson (Eds.), Visualization handbook (pp. 717–731), Butterworth-Heinemann. 10.1016/B978-012387582-2/50038-1

[IMAG.a.1304-b2] Alexander, D. C., Hubbard, P. L., Hall, M. G., Moore, E. A., Ptito, M., Parker, G. J. M., & Dyrby, T. B. (2010). Orientationally invariant indices of axon diameter and density from diffusion MRI. NeuroImage, 52(4), 1374–1389. 10.1016/j.neuroimage.2010.05.043 20580932

[IMAG.a.1304-b3] Andersen, K., Andersen, B. B., & Pakkenberg, B. (2012). Stereological quantification of the cerebellum in patients with Alzheimer’s disease. Neurobiology of Aging, 33(1), 197.e11–197.e20. 10.1016/j.neurobiolaging.2010.06.013 20728248

[IMAG.a.1304-b4] Ashburner, J., & Friston, K. J. (2005). Unified segmentation. NeuroImage, 26(3), 839–851. 10.1016/j.neuroimage.2005.02.018 15955494

[IMAG.a.1304-b5] Avants, B. B., Tustison, N., & Song, G. (2009). Advanced Normalization Tools: V1.0. The Insight Journal, 2(365), 1–35. https://insight-journal.org/browse/publication/681

[IMAG.a.1304-b6] Brouwer, E. J. P., Priovoulos, N., Hashimoto, J., & van der Zwaag, W. (2024). Proprioceptive engagement of the human cerebellum studied with 7T-fMRI. Imaging Neuroscience, 2, imag-2-00268. 10.1162/imag_a_00268 PMC12290657 40800446

[IMAG.a.1304-b7] Brouwer, E. J. P., van der Zwaag, W., Heij, J., & Priovoulos, N. (2025). Easy-to-use b1+ shims for human brain imaging at 7 T. Magnetic Resonance in Medicine, 94(5), 2010–2022. 10.1002/mrm.30617 40554723 PMC12393206

[IMAG.a.1304-b8] Canedo Pouso, L. (2022). Degeneration of the granular cells in the cerebellum in COVID-19 victims (Dissertation). https://urn.kb.se/resolve?urn=urn:nbn:se:uu:diva-485977

[IMAG.a.1304-b9] Chakwizira, A., Zhu, A., Foo, T., Westin, C.-F., Szczepankiewicz, F., & Nilsson, M. (2023). Diffusion MRI with free gradient waveforms on a high-performance gradient system: Probing restriction and exchange in the human brain. NeuroImage, 283, 120409. 10.1016/j.neuroimage.2023.120409 37839729

[IMAG.a.1304-b10] Diedrichsen, J. (2006). A spatially unbiased atlas template of the human cerebellum. NeuroImage, 33(1), 127–138. 10.1016/j.neuroimage.2006.05.056 16904911

[IMAG.a.1304-b11] Diedrichsen, J., Balsters, J. H., Flavell, J., Cussans, E., & Ramnani, N. (2009). A probabilistic MR atlas of the human cerebellum. NeuroImage, 46(1), 39–46. 10.1016/j.neuroimage.2009.01.045 19457380

[IMAG.a.1304-b12] Feinberg, D. A., Beckett, A. J. S., Vu, A. T., Stockmann, J., Huber, L., Ma, S., Ahn, S., Setsompop, K., Cao, X., Park, S., Liu, C., Wald, L. L., Polimeni, J. R., Mareyam, A., Gruber, B., Stirnberg, R., Liao, C., Yacoub, E., Davids, M.,… Dietz, P. (2023). Next-generation MRI scanner designed for ultra-high-resolution human brain imaging at 7 tesla. Nature Methods, 20(12), 2048–2057. 10.1038/s41592-023-02068-7 38012321 PMC10703687

[IMAG.a.1304-b13] Fukutani, Y., Cairns, N. J., Rossor, M. N., & Lantos, P. L. (1996). Purkinje cell loss and astrocytosis in the cerebellum in familial and sporadic Alzheimer’s disease. Neuroscience Letters, 214(1), 33–36. 10.1016/0304-3940(96)12875-5 8873125

[IMAG.a.1304-b14] Giovannucci, A., Badura, A., Deverett, B., Najafi, F., Pereira, T. D., Gao, Z., Ozden, I., Kloth, A. D., Pnevmatikakis, E., Paninski, L., De Zeeuw, C. I., Medina, J. F., & Wang, S. S. (2017). Cerebellar granule cells acquire a widespread predictive feedback signal during motor learning. Nature Neuroscience, 20(5), 727–734. 10.1038/NN.4531 28319608 PMC5704905

[IMAG.a.1304-b15] Grodd, W., Hülsmann, E., Lotze, M., Wildgruber, D., & Erb, M. (2001). Sensorimotor mapping of the human cerebellum: fMRI evidence of somatotopic organization. Human Brain Mapping, 13(2), 55–73. 10.1002/hbm.1025 11346886 PMC6871814

[IMAG.a.1304-b16] Huntenburg, J. M., Steele, C. J., & Bazin, P.-L. (2018). Nighres: Processing tools for high-resolution neuroimaging. GigaScience, 7(7), 082. 10.1093/gigascience/giy082 PMC6065481 29982501

[IMAG.a.1304-b17] JASP Team. (2023). *JASP* (Version 0.18.1) https://jasp-stats.org

[IMAG.a.1304-b18] Jenkinson, M., Beckmann, C. F., Behrens, T. E. J., Woolrich, M. W., & Smith, S. M. (2012). FSL. NeuroImage, 62(2), 782–790. 10.1016/J.NEUROIMAGE.2011.09.015 21979382

[IMAG.a.1304-b19] Kalanjati, V. P., Dewi, A. K., & Santoso, M. W. A. (2017). Quantitative study on human cerebellar cortex from anatomy cadaver preparations. International Journal of Morphology, 35(1), 167–171. 10.4067/S0717-95022017000100027

[IMAG.a.1304-b20] King, M., Hernandez-Castillo, C. R., Poldrack, R. A., Ivry, R. B., & Diedrichsen, J. (2019). Functional boundaries in the human cerebellum revealed by a multi-domain task battery. Nature Neuroscience, 22(8), 1371–1378. 10.1038/s41593-019-0436-x 31285616 PMC8312478

[IMAG.a.1304-b21] Lasič, S., Szczepankiewicz, F., Dall’Armellina, E., Das, A., Kelly, C., Plein, S., Schneider, J., Nilsson, M., & Teh, I. (2020). Motion-compensated b-tensor encoding for in vivo cardiac diffusion-weighted imaging. NMR in Biomedicine, 33(2), e4213. 10.1002/nbm.4213 31765063 PMC6980347

[IMAG.a.1304-b22] Lasič, S., Lundell, H., Szczepankiewicz, F., Nilsson, M., Schneider, J. E., & Teh, I. (2020). Time-dependent and anisotropic diffusion in the heart: Linear and spherical tensor encoding with varying degree of motion compensation. In Proceedings of the 2020 ISMRM & SMRT Virtual Conference & Exhibition (pp. 1–3). Abstract number: 4300. https://archive.ismrm.org/2020/4300.html

[IMAG.a.1304-b24] Manto, M.-U. (2005). The wide spectrum of spinocerebellar ataxias (SCAs). The Cerebellum, 4(1), 2–6. 10.1080/14734220510007914 15895552

[IMAG.a.1304-b25] Marques, J. P., van der Zwaag, W., Granziera, C., Krueger, G., & Gruetter, R. (2010). Cerebellar cortical layers: In vivo visualization with structural high-field-strength MR imaging. Radiology, 254(3), 942–948. 10.1148/radiol.09091136 20177104

[IMAG.a.1304-b26] Martin, J., Endt, S., Wetscherek, A., Kuder, T. A., Doerfler, A., Uder, M., Hensel, B., & Laun, F. B. (2020). Contrast-to-noise ratio analysis of microscopic diffusion anisotropy indices in q-space trajectory imaging. Zeitschrift für Medizinische Physik, 30(1), 4–16. 10.1016/j.zemedi.2019.01.003 30853147

[IMAG.a.1304-b27] Mori, S., & Van Zijl, P. C. M. (1995). Diffusion weighting by the trace of the diffusion tensor within a single scan. Magnetic Resonance in Medicine, 33(1), 41–52. 10.1002/mrm.1910330107 7891534

[IMAG.a.1304-b28] Nettekoven, C., Zhi, D., Shahshahani, L., Pinho, A. L., Saadon-Grosman, N., Buckner, R. L., & Diedrichsen, J. (2024). A hierarchical atlas of the human cerebellum for functional precision mapping. Nature Communications, 15(1), 8376. 10.1038/s41467-024-52371-w PMC11436828 39333089

[IMAG.a.1304-b29] Nilsson, M., Eklund, G., Szczepankiewicz, F., Skorpil, M., Bryskhe, K., Westin, C.-F., Lindh, C., Blomqvist, L., & Jäderling, F. (2021). Mapping prostatic microscopic anisotropy using linear and spherical b-tensor encoding: A preliminary study. Magnetic Resonance in Medicine, 86(4), 2025–2033. 10.1002/mrm.28856 34056750 PMC9272946

[IMAG.a.1304-b30] Nilsson, M., Englund, E., Szczepankiewicz, F., van Westen, D., & Sundgren, P. C. (2018). Imaging brain tumour microstructure. NeuroImage, 182, 232–250. 10.1016/j.neuroimage.2018.04.075 29751058

[IMAG.a.1304-b31] Norman, R. M. (1940). Primary degeneration of the granular layer of the cerebellum: An unusual form of the familial cerebellar atrophy occureing in early life. Brain, 63(4), 365–379. 10.1093/brain/63.4.365

[IMAG.a.1304-b32] Norris, D. G., Koopmans, P. J., Boyacioğlu, R., & Barth, M. (2011). Power independent of number of slices (PINS) radiofrequency pulses for low-power simultaneous multislice excitation. Magnetic Resonance in Medicine, 66(5), 1234–1240. 10.1002/mrm.23152 22009706

[IMAG.a.1304-b33] Priovoulos, N., Andersen, M., Dumoulin, S. O., Boer, V. O., & van der Zwaag, W. (2023). High-resolution motion-corrected 7.0-t MRI to derive morphologic measures from the human cerebellum in vivo. Radiology, 307(2), e220989. 10.1148/RADIOL.220989 36648348

[IMAG.a.1304-b34] Priovoulos, N., & Bazin, P.-L. (2023). Methods for cerebellar imaging analysis. Current Opinion in Behavioral Sciences, 54, 101328. 10.1016/j.cobeha.2023.101328

[IMAG.a.1304-b35] Rusholt, E. H. L., Salvesen, L., Brudek, T., Tesfay, B., Pakkenberg, B., & Olesen, M. V. (2020). Pathological changes in the cerebellum of patients with multiple system atrophy and parkinson’s disease—A stereological study. Brain Pathology, 30(3), 576–588. 10.1111/bpa.12806 31769073 PMC8018044

[IMAG.a.1304-b36] Schmahmann, J. D., MacMore, J., & Vangel, M. (2009). Cerebellar stroke without motor deficit: Clinical evidence for motor and non-motor domains within the human cerebellum. Neuroscience, 162(3), 852–861. 10.1016/j.neuroscience.2009.06.023 19531371 PMC2763197

[IMAG.a.1304-b37] Sereno, M. I., Diedrichsen, J., Tachrount, M., Testa-Silva, G., d’Arceuil, H., & De Zeeuw, C. (2020). The human cerebellum has almost 80% of the surface area of the neocortex. Proceedings of the National Academy of Sciences of the United States of America, 117(32), 19538–19543. 10.1073/pnas.2002896117 32723827 PMC7431020

[IMAG.a.1304-b38] Shilling, R. Z., Robbie, T. Q., Bailloeul, T., Mewes, K., Mersereau, R. M., & Brummer, M. E. (2009). A super-resolution framework for 3-D high-resolution and high-contrast imaging using 2-D multislice MRI. IEEE Transactions on Medical Imaging, 28(5), 633–644. 10.1109/TMI.2008.2007348 19272995

[IMAG.a.1304-b39] Sjölund, J., Szczepankiewicz, F., Nilsson, M., Topgaard, D., Westin, C.-F., & Knutsson, H. (2015). Constrained optimization of gradient waveforms for generalized diffusion encoding. Journal of Magnetic Resonance, 261, 157–168. 10.1016/j.jmr.2015.10.012 26583528 PMC4752208

[IMAG.a.1304-b40] Stoodley, C. J., & Schmahmann, J. D. (2009). Functional topography in the human cerebellum: A meta-analysis of neuroimaging studies. NeuroImage, 44(2), 489–501. 10.1016/j.neuroimage.2008.08.039 18835452

[IMAG.a.1304-b41] Szczepankiewicz, F., Sjölund, J., Ståhlberg, F., Lätt, J., & Nilsson, M. (2019). Tensor-valued diffusion encoding for diffusional variance decomposition (DIVIDE): Technical feasibility in clinical MRI systems. PLoS One, 14(3), 0214238. 10.1371/journal.pone.0214238 PMC6438503 30921381

[IMAG.a.1304-b42] Szczepankiewicz, F., van Westen, D., Englund, E., Westin, C.-F., Ståhlberg, F., Lätt, J., Sundgren, P. C., & Nilsson, M. (2016). The link between diffusion MRI and tumor heterogeneity: Mapping cell eccentricity and density by diffusional variance decomposition (DIVIDE). NeuroImage, 142, 522–532. 10.1016/j.neuroimage.2016.07.038 27450666 PMC5159287

[IMAG.a.1304-b43] Szczepankiewicz, F., Westin, C.-F., & Nilsson, M. (2019). Maxwell-compensated design of asymmetric gradient waveforms for tensor-valued diffusion encoding. Magnetic Resonance in Medicine, 82(4), 1424–1437. 10.1002/mrm.27828 31148245 PMC6626569

[IMAG.a.1304-b44] Szczepankiewicz, F., Westin, C.-F., & Nilsson, M. (2021). Gradient waveform design for tensor-valued encoding in diffusion MRI. Journal of Neuroscience Methods, 348, 109007. 10.1016/j.jneumeth.2020.109007 33242529 PMC8443151

[IMAG.a.1304-b45] Tallantyre, E., Dixon, J., Donaldson, I., Owens, T., Morgan, P., Morris, P., & Evangelou, N. (2011). Ultra-high-field imaging distinguishes MS lesions from asymptomatic white matter lesions. Neurology, 76(6), 534–539. 10.1212/WNL.0b013e31820b7630 21300968 PMC3053180

[IMAG.a.1304-b46] Tax, C. M. W., Genc, S., MacIver, C. L., Nilsson, M., Wardle, M., Szczepankiewicz, F., Jones, D. K., & Peall, K. J. (2023). Ultra-strong diffusion-weighted MRI reveals cerebellar grey matter abnormalities in movement disorders. NeuroImage: Clinical, 38, 103419. 10.1016/j.nicl.2023.103419 37192563 PMC10199248

[IMAG.a.1304-b47] Tax, C. M. W., Szczepankiewicz, F., Nilsson, M., & Jones, D. K. (2020). The dot-compartment revealed? diffusion MRI with ultra-strong gradients and spherical tensor encoding in the living human brain. NeuroImage, 210, 116534. 10.1016/j.neuroimage.2020.116534 31931157 PMC7429990

[IMAG.a.1304-b48] ten Donkelaar, H. J., den Dunnen, W., Lammens, M., Wesseling, P., Willemsen, M., & Hori, A. (2023). Development and developmental disorders of the human cerebellum. Journal of Neurology, 250(9), 1025–36. 10.1007/978-3-031-26098-8_8 14504962

[IMAG.a.1304-b49] Vachha, B., & Huang, S. Y. (2021). MRI with ultrahigh field strength and high-performance gradients: Challenges and opportunities for clinical neuroimaging at 7 T and beyond. European Radiology Experimental, 5(1), 35. 10.1186/s41747-021-00216-2 34435246 PMC8387544

[IMAG.a.1304-b50] van der Zwaag, W., Kusters, R., Magill, A., Gruetter, R., Martuzzi, R., Blanke, O., & Marques, J. P. (2013). Digit somatotopy in the human cerebellum: A 7T fMRI study. NeuroImage, 67, 354–362. 10.1016/j.neuroimage.2012.11.041 23238433

[IMAG.a.1304-b51] Versteeg, E., van der Velden, T. A., van Leeuwen, C. C., Borgo, M., Huijing, E. R., Hendriks, A. D., Hendrikse, J., Klomp, D. W. J., & Siero, J. C. W. (2021). A plug-and-play, lightweight, single-axis gradient insert design for increasing spatiotemporal resolution in echo planar imaging-based brain imaging. NMR in Biomedicine, 34(6), e4499. 10.1002/nbm.4499 33619838 PMC8244051

[IMAG.a.1304-b52] Vis, G., Nilsson, M., Westin, C. F., & Szczepankiewicz, F. (2021). Accuracy and precision in super-resolution MRI: Enabling spherical tensor diffusion encoding at ultra-high b-values and high resolution. NeuroImage, 245, 118673. 10.1016/J.NEUROIMAGE.2021.118673 34688898 PMC9272945

[IMAG.a.1304-b53] Wegiel, J., Wisniewski, H. M., Dziewiatkowski, J., Badmajew, E., Tarnawski, M., Reisberg, B., Mlodzik, B., De Leon, M. J., & Miller, D. C. (1999). Cerebellar atrophy in Alzheimer’s disease—Clinicopathological correlations. Brain Research, 818(1), 41–50. 10.1016/S0006-8993(98)01279-7 9914436

[IMAG.a.1304-b54] Wong, E. C., Cox, R. W., & Song, A. W. (1995). Optimized isotropic diffusion weighting. Magnetic Resonance in Medicine, 34(2), 139–143. 10.1002/mrm.1910340202 7476070

[IMAG.a.1304-b55] Zheng, J., Yang, Q., Makris, N., Huang, K., Liang, J., Ye, C., Yu, X., Tian, M., Ma, T., Mou, T., Guo, W., Kikinis, R., & Gao, Y. (2022). Three-dimensional digital reconstruction of the cerebellar cortex: Lobule thickness, surface area measurements, and layer architecture. Cerebellum, 22(2), 249–260. 10.1007/s12311-022-01390-8 35286708 PMC9470778

