# Supplementary material for: Human cerebellar microstructure with 7T MRI: Spherical b-tensor encoding and super resolution reconstruction
Source: Imaging Neurosci (Camb). 2026 Aug 12;4:IMAG.a.1304. doi: 10.1162/IMAG.a.1304 (PMC13471089; doi:10.1162/IMAG.a.1304)
Supplement: Supplementary file 1 [file IMAG.a.1304_supp.pdf]

Supplementary Material

Participant	Cer GM	Cer WM	Vis GM	Vis WM
1	83.89	59.76	84.07	64.10
2	61.75	55.48	60.84	40.36
3	68.90	56.09	82.97	61.67
4	62.30	58.19	52.22	33.88
5	86.90	71.19	57.46	49.76
6	69.00	56.19	94.22	60.38
7	73.69	54.45	57.69	39.70
8	89.87	68.11	87.42	60.98
9	70.92	64.79	56.46	45.20
10	60.42	56.72	52.43	37.96
11	75.52	64.24	61.37	45.27
**Mean**	73.01	60.47	67.92	49.02
**SD**	10.20	5.70	15.76	10.96

Table S1: The median diffusion weighted signal for b=0s/mm^2 across four ROIs in the grey and white matter of cerebellum and visual cortex. Cer=Cerebellum, Vis=Visual Cortex, GM=Grey Matter, WM=White Matter

Participant	Cer GM	Cer WM	Vis GM	Vis WM
1	29.37	24.44	22.88	21.92
2	22.15	23.23	12.72	12.47
3	25.18	24.99	26.24	24.89
4	24.50	24.26	12.88	11.27
5	32.40	30.20	18.97	18.35
6	25.81	23.94	23.79	22.24
7	21.61	21.03	12.56	12.09
8	31.13	28.61	21.78	20.79
9	29.35	26.24	19.26	17.96
10	24.99	23.75	15.30	14.14
11	28.64	26.65	18.89	17.47
**Mean**	26.83	25.21	18.66	17.60
**SD**	3.57	2.57	4.79	4.61

Table S2: The median diffusion weighted signal for b=$1000 s/mm^2$ across four ROIs in the grey and white matter of cerebellum and visual cortex. Cer=Cerebellum, Vis=Visual Cortex, GM=Grey Matter, WM=White Matter

Participant	Cer GM	Cer WM	Vis GM	Vis WM
1	5.95	4.49	3.44	3.52
2	4.57	4.11	2.28	2.36
3	5.54	4.72	3.97	4.02
4	4.85	3.62	2.11	2.06
5	6.49	5.02	3.04	3.03
6	5.86	4.62	3.76	3.73
7	4.30	3.54	1.99	2.05
8	6.64	5.77	3.51	3.57
9	5.92	4.36	3.11	3.03
10	5.06	4.00	2.48	2.43
11	5.79	4.57	2.97	2.91
**Mean**	5.54	4.44	2.97	2.98
**SD**	0.76	0.63	0.68	0.69

Table S3: The median diffusion weighted signal for b=3500 s/mm^2 across four ROIs in the grey and white matter of cerebellum and visual cortex. Cer=Cerebellum, Vis=Visual Cortex, GM=Grey Matter, WM=White Matter

Statistical Comparison	Signal b0		Signal b1000		Signal b3500	
	p value	t stat	p value	t stat	p value	t stat
Grey matter vs White matter	<**0.001****	9.25	<**0.001****	4.96	<**0.001****	9.49
Cerebellum vs Visual Cortex	**0.05***	2.19	<**0.001****	6.28	<**0.001****	14.52
Cer GM vs Vis GM	0.352	1.13	<**0.001****	6.43	<**0.001****	16.72
Cer GM vs Cer WM	<**0.001****	1.13	**0.009***	3.27	<**0.001****	16.72
Cer GM vs Vis WM	<**0.001****	8.38	<**0.001****	7.49	<**0.001****	15.99
Vis GM vs Cer WM	0.35	1.46	0.001*	-4.9	<**0.001****	-10.49
Vis GM vs Vis WM	<**0.001****	8.85	<**0.001****	7.80	0.812	-0.24
Cer WM vs Vis WM	**0.021***	3.37	<**0.001****	5.91	<**0.001****	10.51

Table S4: Statistical comparison of median diffusion weighted signals across different ROIs using a repeated measures Anova with post hoc comparisons. * indicate statistical significance (* $p < 0.05$, ** $p < 0.01$).

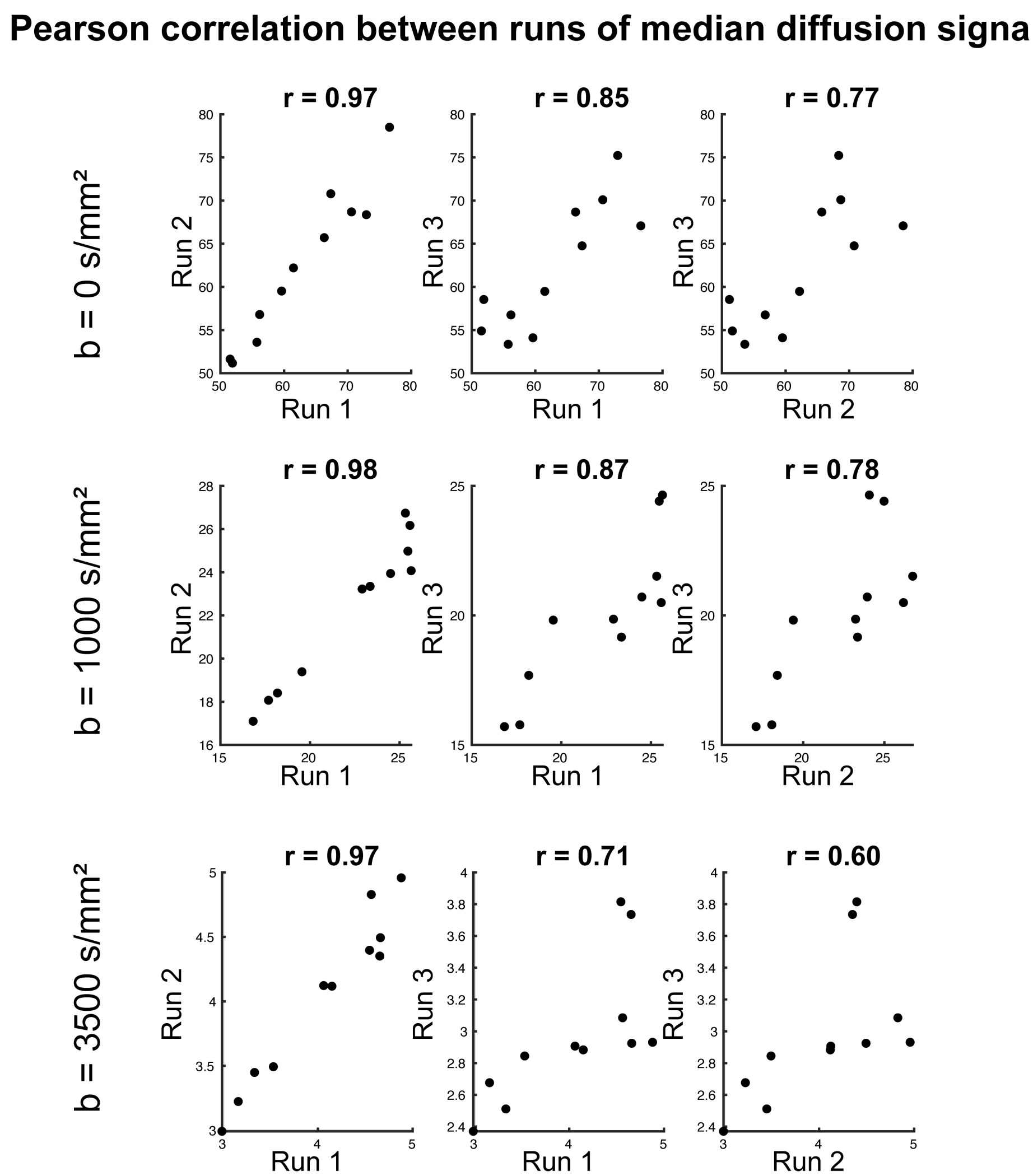

Figure S1: The Pearson correlations (r) between the diffusion weighted signal in run 1,2 and 3 (the median signal is averaged across four segmentations: cerebellar/visual cortex grey/white matter).

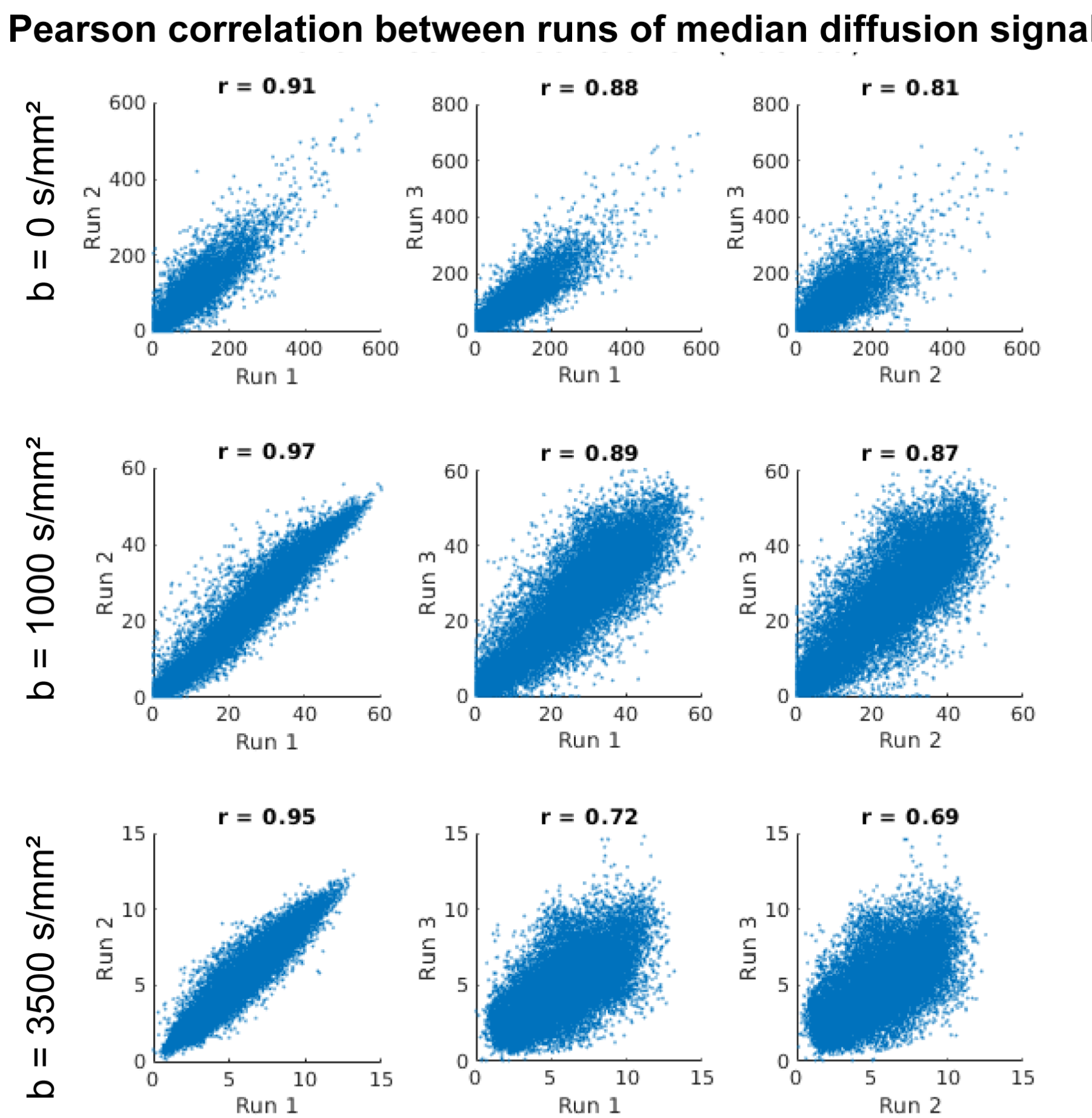

Figure S2: The Pearson correlations (r) between the diffusion weighted signal of participant 1 in run 1-3 for each voxel.

Participant	Cer GM	Vis GM
1	0.37	0.28
2	0.37	0.20
3	0.39	0.31
4	0.39	0.25
5	0.38	0.35
6	0.40	0.25
7	0.28	0.21
8	0.35	0.25
9	0.43	0.34
10	0.42	0.30
11	0.39	0.31
**Mean**	0.38	0.28
**SD**	0.04	0.05
**p-value**	<0.001	
**t-stat**	8.40	

Table S5: The normalised diffusion weighted signal for b=1000s/mm^2 across two ROIs in the grey matter of cerebellum and visual cortex. Cer=Cerebellum, Vis=Visual Cortex, GM=Grey Matte. The bottom two rows present the results (p value and t stat) of a paired samples t-test.

Participant	Cer GM	Vis GM
1	0.08	0.04
2	0.08	0.04
3	0.09	0.05
4	0.08	0.04
5	0.08	0.05
6	0.09	0.04
7	0.06	0.04
8	0.08	0.04
9	0.09	0.06
10	0.09	0.05
11	0.08	0.05
**Mean**	0.08	0.04
**SD**	0.01	0.00
**p-value**	<0.001	
**t-stat**	14.91	

Table S6: The normalised diffusion weighted signal for b=3500s/mm^2 across two ROIs in the grey matter of cerebellum and visual cortex. Cer=Cerebellum, Vis=Visual Cortex, GM=Grey Matter. The bottom two rows present the results (p value and t stat) of a paired samples t-test.

Lobule	p1	p2	p3	p4	p5	p6	p7	p8	p9	p10	p11	Mean	SD
**Anterior Lobe:**													
Left I–IV	0.06	0.06	0.07	0.05	0.05	0.05	0.04	0.05	0.06	0.06	0.06	0.06	0.01
Right I–IV	0.06	0.06	0.07	0.06	0.06	0.07	0.05	0.06	0.06	0.07	0.06	0.06	0.01
Left V	0.06	0.07	0.06	0.06	0.05	0.07	0.04	0.05	0.07	0.06	0.06	0.06	0.01
Right V	0.06	0.06	0.07	0.06	0.06	0.07	0.04	0.06	0.07	0.07	0.06	0.06	0.01
**Posterior Lobe:**													
Left VI	0.07	0.08	0.08	0.08	0.07	0.09	0.05	0.07	0.08	0.08	0.08	0.07	0.01
Vermis VI	0.06	0.05	0.05	0.05	0.05	0.05	0.03	0.04	0.07	0.06	0.05	0.05	0.01
Right VI	0.08	0.08	0.08	0.08	0.07	0.09	0.06	0.07	0.08	0.08	0.07	0.07	0.01
Left Crus I	0.06	0.07	0.08	0.07	0.08	0.08	0.04	0.07	0.08	0.08	0.08	0.07	0.01
Vermis Crus I	0.06	0.03	0.08	0.04	0.05	0.09	0.01	0.02	0.06	NaN	0.03	0.05	0.03
Right Crus I	0.07	0.07	0.08	0.07	0.07	0.08	0.05	0.07	0.09	0.08	0.07	0.07	0.01
Left Crus II	0.08	0.07	0.08	0.08	0.08	0.09	0.07	0.07	0.09	0.09	0.08	0.08	0.01
Vermis Crus II	0.05	0.06	0.04	0.04	0.06	0.05	0.03	0.04	0.07	0.05	0.03	0.05	0.01
Right Crus II	0.08	0.08	0.08	0.08	0.09	0.09	0.06	0.07	0.09	0.09	0.08	0.08	0.01
Left VIIb	0.08	0.08	0.08	0.08	0.08	0.09	0.07	0.09	0.09	0.08	0.09	0.09	0.01
Vermis VIIb	0.06	0.05	0.08	0.05	0.08	0.06	0.06	0.06	0.07	0.05	0.07	0.06	0.01
Right VIIb	0.09	0.09	0.10	0.09	0.09	0.11	0.08	0.10	0.10	0.09	0.09	0.09	0.01
Left VIIIa	0.08	0.09	0.09	0.09	0.09	0.09	0.08	0.08	0.08	0.08	0.09	0.09	0.01
Vermis VIIIa	0.08	0.07	0.07	0.07	0.07	0.08	0.04	0.07	0.08	0.08	0.08	0.07	0.01
Right VIIIa	0.10	0.10	0.11	0.09	0.10	0.13	0.09	0.10	0.10	0.09	0.10	0.10	0.01
Left VIIIb	0.08	0.08	0.09	0.08	0.09	0.08	0.07	0.08	0.08	0.08	0.08	0.08	0.01
Vermis VIIIb	0.07	0.05	0.08	0.05	0.06	0.06	0.07	0.07	0.08	0.05	0.07	0.07	0.01
Right VIIIb	0.10	0.09	0.10	0.08	0.09	0.12	0.08	0.09	0.08	0.09	0.09	0.09	0.01
Left IX	0.07	0.07	0.09	0.07	0.05	0.05	0.05	0.06	0.07	0.08	0.06	0.06	0.01
Vermis IX	0.08	0.08	0.08	0.07	0.08	0.08	0.06	0.07	0.08	0.08	0.07	0.08	0.01
Right IX	0.08	0.08	0.09	0.07	0.07	0.09	0.05	0.07	0.07	0.08	0.07	0.07	0.01
Left X	0.02	0.04	0.05	0.03	0.03	0.01	0.03	0.05	0.04	0.04	0.04	0.04	0.01
Vermis X	0.05	0.05	0.08	0.03	0.04	0.02	0.03	0.03	0.04	0.06	0.03	0.04	0.01
Right X	0.05	0.04	0.05	0.04	0.05	0.07	0.03	0.03	0.06	0.04	0.04	0.04	0.01
Dentate (L)	0.07	0.08	0.09	0.07	0.08	0.08	0.07	0.08	0.08	0.07	0.09	0.08	0.01
Dentate (R)	0.07	0.08	0.09	0.07	0.08	0.08	0.07	0.08	0.08	0.07	0.09	0.08	0.01

Table S7: The normalised diffusion weighted signal for b=3500 s/mm^2 across 30 different cerebellar ROIs. p indicates the participant number, SD=standard deviation across participants. The top four rows form the anterior lobe, rows 5-28 form the posterior lobe and the last two rows the left and right dentate nucleus.

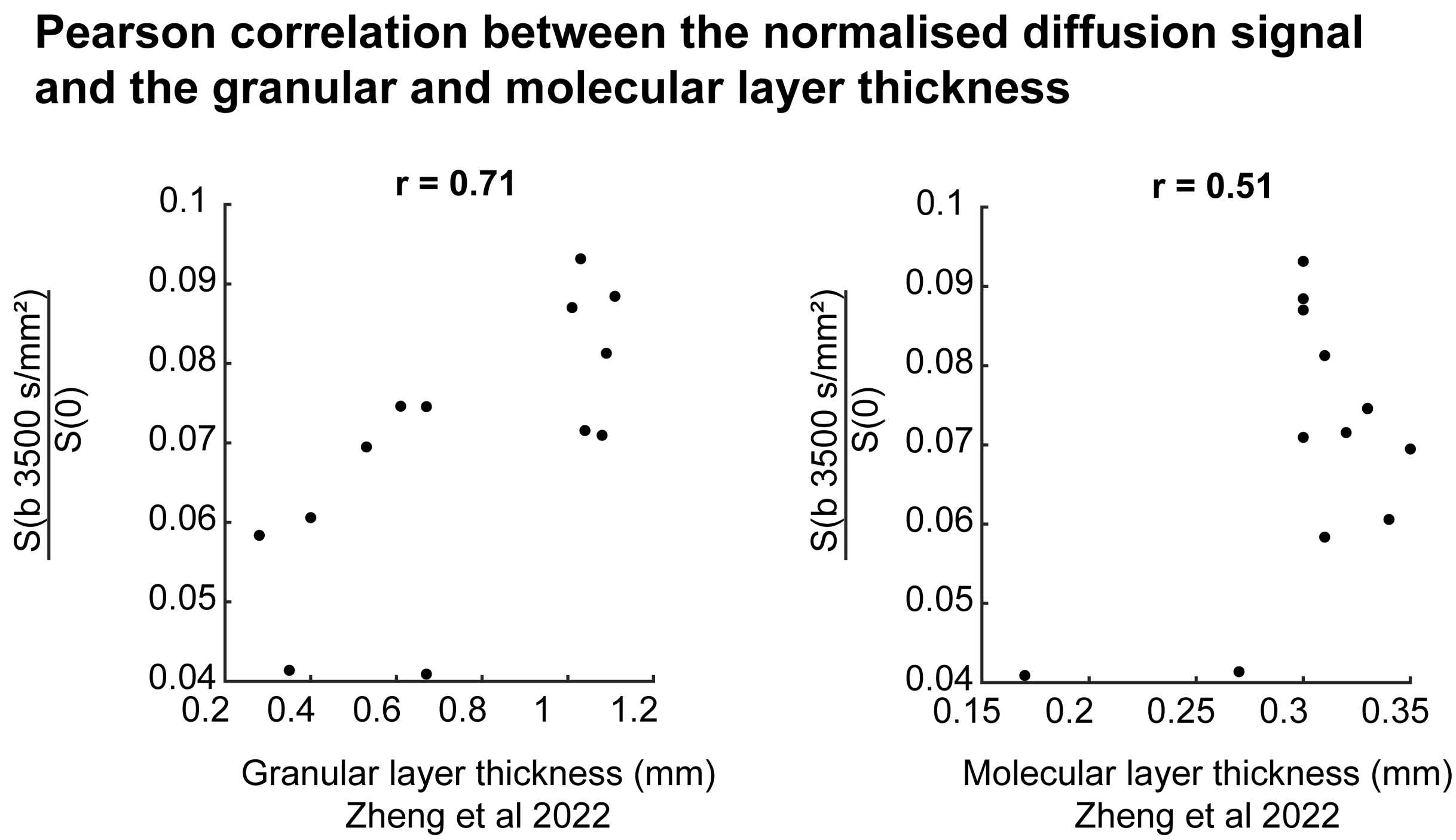

Figure S3: We computed the Pearson correlation between the group-averaged values of measure of the normalised diffusion weighted signal (b=$3500s/mm^2$) across cerebellar lobules and the corresponding post mortem values of the granular layer thickness (Zheng et al., 2022). The same correlation was performed for the corresponding post mortem values of the molecular layer thickness (Zheng et al., 2022).

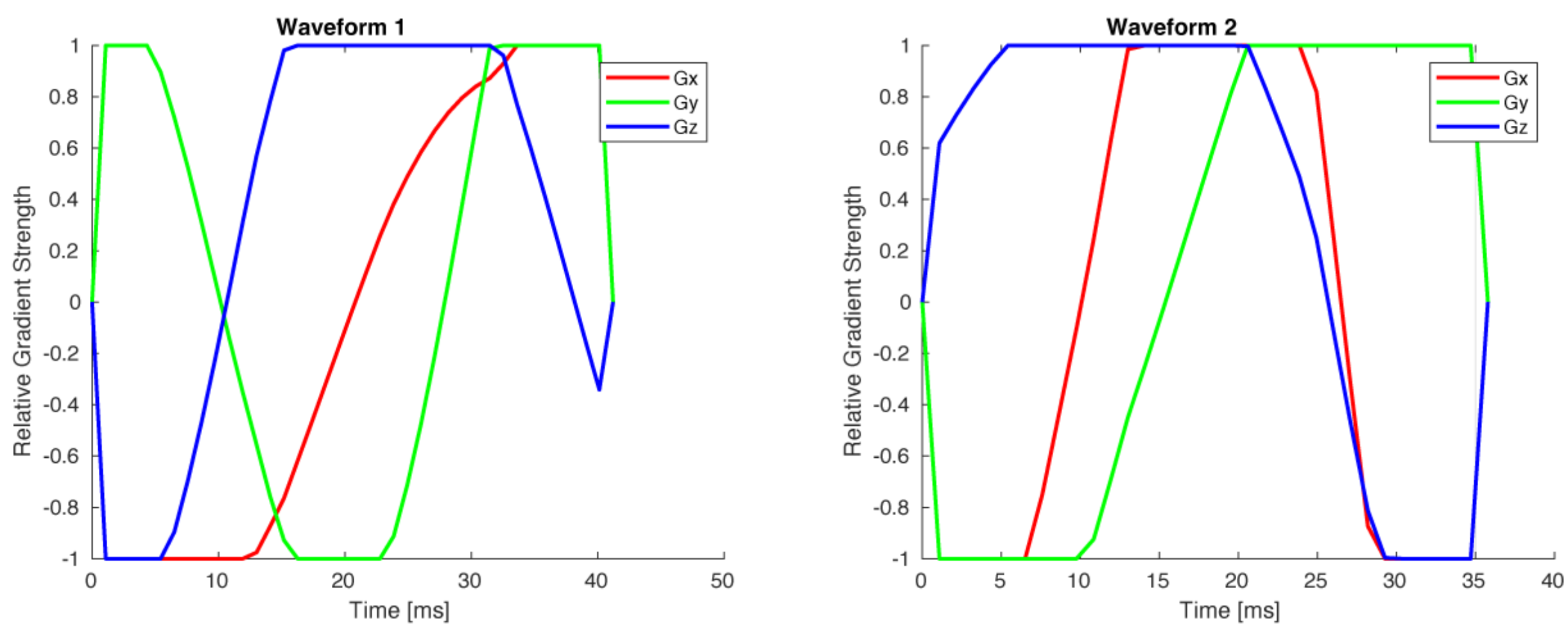

Figure S4: The gradient waveforms used for the diffusion acquisition. Waveform 1 takes place before the 180 degree pulse, waveform 2 takes place after the 180 degree pulse.
